# Supplementary material for: Loss‐of‐function variants in Kv11.1 cardiac channels as a biomarker for SUDEP
Source: Ann Clin Transl Neurol. 2021 May 18;8(7):1422–32. doi: 10.1002/acn3.51381 (PMC8283159; doi:10.1002/acn3.51381)
Supplement: Supplementary file 1 — Table S1. Characteristics of SUDEP patients without KCNH2 variant (orange‐shaded patients carried loss‐of‐function KCNH2 variants). Table S2. Characteristics of control epilepsy patients with KCNH2 variant (orange‐shaded patients carried loss‐of‐function KCNH2 variants). Table S3. Types of epilepsies in the control cohort. [file ACN3-8-1422-s001.docx]

**Supplementary Table 1**: Characteristics of SUDEP patients without *KCNH2* variant (orange-shaded patients carried loss-of-function *KCNH2* variants)

| **Age died** | **Epilepsy type*** | **AED postmortem** | ***KCNH2* variant** | **Functional impact** |
| --- | --- | --- | --- | --- |
| 23 | Epilepsy unclassified | Valproic acid | R1047L | Loss-of-function |
| 59 | Epilepsy unclassified | None detected | R1047L | Loss-of-function |
| 52 | Epilepsy unclassified | None detected | R1047L | Loss-of-function |
| 52 | Epilepsy unclassified | Subtherapeutic level valproic acid | R1047L | Loss-of-function |
| 28 | Non-lesional focal epilepsy: temporal | Carbamazepine | R1047L | Loss-of-function |
| 39 | Epilepsy unclassified | Phenytoin | R176W | No change |
| 40 | Lesional focal epilepsy: temporal | Lamotrigine | R744X | Loss-of-function |
| 25 | Epilepsy unclassified | Carbamazepine | G749A | Loss-of-function |
| 36 | Lesional focal epilepsy: temporal: hippocampal sclerosis; post-meningitis | Lamotrigine | G924A | Loss-of-function |
| 25 | Epilepsy unclassified | None detected | Y54H | No change |
| 51 | Epilepsy unclassified | Lamotrigine, phenytoin |  |  |
| 21 | Epilepsy unclassified | Valproic acid |  |  |
| 20 | Epilepsy unclassified | None detected |  |  |
| 29 | Lesional focal epilepsy: unspecified focus | Lamotrigine |  |  |
| 29 | Epilepsy unclassified | Phenytoin |  |  |
| 21 | Epilepsy unclassified | None detected |  |  |
| 21 | Epilepsy unclassified | Carbamazepine |  |  |
| 28 | Epilepsy unclassified | None detected |  |  |
| 20 | Epilepsy unclassified | Carbamazepine |  |  |
| 29 | Epilepsy unclassified | None detected |  |  |
| 35 | Epilepsy unclassified | Phenytoin |  |  |
| 21 | Epilepsy unclassified | None detected |  |  |
| 60 | Epilepsy unclassified | Subtherapeutic level |  |  |
| 72 | Epilepsy unclassified | Therapeutic level |  |  |
| 19 | Epilepsy unclassified | None detected |  |  |
| 23 | Epilepsy unclassified | Subtherapeutic level sodium valproate & therapeutic level lamotrigine |  |  |
| 35 | Epilepsy unclassified | No data |  |  |
| 48 | Epilepsy unclassified | No data |  |  |
| 42 | Epilepsy unclassified | Phenytoin |  |  |
| 42 | Epilepsy unclassified | Valproic acid |  |  |
| 56 | Epilepsy unclassified | No data |  |  |
| 41 | Epilepsy unclassified | Therapeutic level phenytoin |  |  |
| 42 | Epilepsy unclassified | Subtherapeutic level phenytoin |  |  |
| 53 | Epilepsy unclassified | Phenytoin, valproic acid |  |  |
| 36 | Epilepsy unclassified | None detected |  |  |
| 44 | Epilepsy unclassified | Lamotrigine, oxcarbazepine |  |  |
| 36 | Epilepsy unclassified | No data |  |  |
| 29 | Epilepsy unclassified | Therapeutic level carbamazepine and lamotrigine |  |  |
| 34 | Epilepsy unclassified | No data |  |  |
| 17 | Epilepsy unclassified | Therapeutic level AED |  |  |
| 56 | Epilepsy unclassified | Subtherapeutic level valproic acid |  |  |
| 30 | Lesional focal epilepsy: fronto-temporal | Carbamazepine |  |  |
| 38 | Epilepsy unclassified | No data |  |  |
| 34 | Epilepsy unclassified | Phenytoin |  |  |
| 34 | Epilepsy unclassified | Carbamazepine |  |  |
| 45 | Epilepsy unclassified | No data |  |  |
| 31 | Epilepsy unclassified | Carbamazepine |  |  |
| 21 | Epilepsy unclassified | Lamotrigine |  |  |
| 12 | Epilepsy unclassified | No data |  |  |
| 31 | Epilepsy unclassified | Therapeutic level lamotrigine |  |  |
| 40 | Epilepsy unclassified | Oxcarbazepine |  |  |
| 48 | Lesional focal epilepsy: traumatic brain injury | None detected |  |  |
| 25 | Epilepsy unclassified | Subtherapeutic level valproic acid |  |  |
| 82 | Epilepsy unclassified | Therapeutic level valproic acid |  |  |
| 26 | Epilepsy unclassified | None detected |  |  |
| 44 | Epilepsy unclassified | Subtherapeutic level carbamazepine and phenytoin |  |  |
| 41 | Epilepsy unclassified | Subtherapeutic level lamotrigine and valproate |  |  |
| 79 | Epilepsy unclassified | Subtherapeutic level valproic acid |  |  |
| 46 | Epilepsy unclassified | No data |  |  |
| 43 | Epilepsy unclassified | Therapeutic level carbamazepine |  |  |
| 28 | Epilepsy unclassified | Carbamazepine |  |  |
| 25 | Epilepsy unclassified | Valproic acid |  |  |
| 43 | Epilepsy unclassified | No data |  |  |
| 25 | Lesional focal epilepsy: fronto-temporal dysplasia | No data |  |  |
| 16 | Combined generalised and focal epilepsy | No data |  |  |
| 19 | Lesional focal epilepsy: dysplasia | No data |  |  |
| 7 | Developmental and Epileptic Encephalopathy: Dravet syndrome | No data |  |  |
| 28 | Genetic generalised epilepsy: Juvenile myoclonic epilepsy | No data |  |  |
| 40 | Genetic generalised epilepsy: Juvenile absence epilepsy | Valproic acid |  |  |
| 31 | Lesional focal epilepsy: temporal: hippocampal sclerosis | Carbamazepine |  |  |
| 9 | Epilepsy unclassified | No data |  |  |
| 18 | Genetic generalised epilepsy: Juvenile myoclonic epilepsy | No data |  |  |
| 49 | Genetic generalised epilepsy: Childhood absence epilepsy | Carbamazepine, lamotrigine, phenytoin |  |  |
| 23 | Non-lesional focal epilepsy: unspecified focus | No data |  |  |
| 2 | Epileptic Encephalopathy | No data |  |  |
| 20 | Epileptic Encephalopathy | No data |  |  |
| 37 | Lesional focal epilepsy: malformation: schizencephaly | Carbamazepine |  |  |
| 1 | Epileptic Encephalopathy | No data |  |  |
| 1 | Epileptic Encephalopathy | Clonazepam, phenytoin |  |  |
| 36 | Non-lesional focal epilepsy: frontal | No data |  |  |
| 9 | Myoclonic-atonic epilepsy | Clobazam |  |  |
| 31 | Genetic generalised epilepsy: Juvenile myoclonic epilepsy | No data |  |  |
| 38 | Structural focal epilepsy: malformation: polymicrogyria | None detected |  |  |
| 19 | Combined generalised and focal epilepsy | No data |  |  |
| 43 | Epilepsy unclassified | No data |  |  |
| 30 | Generalised epilepsy: malformation: subcortical band heterotopia | None detected |  |  |
| 24 | Non-lesional focal epilepsy: fronto-temporal | Lamotrigine, topiramate |  |  |
| 45 | Lesional focal epilepsy: dysplasia | No data |  |  |
| 20 | Non-lesional focal epilepsy: unspecified | None detected |  |  |
| 21 | Epilepsy unclassified | Lamotrigine |  |  |

*Unclassified cases generally due IRB restrictions from one jurisdiction on obtaining clinical data

**Supplementary Table 2**: Characteristics of control epilepsy patients with *KCNH2* variant (orange-shaded patients carried loss-of-function *KCNH2* variants)

| **Age** | **Epilepsy type** | ***KCNH2* variant** | **Functional impact** |
| --- | --- | --- | --- |
| 59 | Non-lesional focal epilepsy: temporal | R1047L | Loss-of-function |
| 60 | Non-lesional focal epilepsy: temporal | R1047L | Loss-of-function |
| 90 | Non-lesional focal epilepsy: temporal | R1047L | Loss-of-function |
| 54 | Non-lesional focal epilepsy: unspecified | R1047L | Loss-of-function |
| 57 | Non-lesional focal epilepsy: unspecified | R1047L | Loss-of-function |
| 56 | Lesional: structural focal epilepsy: benign tumour | R1047L | Loss-of-function |
| 61 | Lesional: structural focal epilepsy: stroke | R1047L | Loss-of-function |
| 55 | Genetic generalised epilepsy: generalised unspecified | R1047L | Loss-of-function |
| 74 | Focal: other non-lesional focal epilepsy: temporal | R1047L | Loss-of-function |
| 55 | Focal: other non-lesional focal epilepsy: occipital | R1047L | Loss-of-function |
| 69 | Structural focal epilepsy: vascular malformation: cerebral angioma | A913V | No change |
| 70 | Non-lesional focal epilepsy: temporal | G903R | No change |
| 68 | Lesional: structural focal epilepsy: traumatic brain injury | K897R | No change |
| 62 | Non-lesional focal epilepsy: temporal | S871C | No change |
| 66 | Non-lesional focal epilepsy: frontal | T436M | No change |
| 56 | Non-lesional focal epilepsy: frontal | R397H | No change |
| 69 | Genetic generalised epilepsy: febrile seizures only | P347S | No change |
| 64 | Genetic generalised epilepsy: febrile seizures plus/genetic epilepsy with febrile seizures plus | D259N | No change |
| 88 | Non-lesional focal epilepsy: frontal | A193V | No change |
| 57 | Non-lesional focal epilepsy: unspecified | S140F | Loss-of-function |

**Supplementary Table 3**: Types of epilepsies in control cohort

| **Epilepsy types** | **Proportion of patients** |
| --- | --- |
| Developmental and Epileptic Encephalopathies: Dravet syndrome | 0.3 |
| Developmental and Epileptic Encephalopathies: Epilepsy with myoclonic atonic seizures | 0.6 |
| Developmental and Epileptic Encephalopathies: Lennox-Gastaut syndrome | 2.1 |
| Developmental and Epileptic Encephalopathies: Nonsyndromic epileptic encephalopathy with focal seizures | 0.3 |
| Developmental and Epileptic Encephalopathies: Nonsyndromic epileptic encephalopathy with generalized seizures | 0.6 |
| Lesional: Focal unspecified | 0.3 |
| Other non-lesional focal epilepsies: Frontal | 3.3 |
| Other non-lesional focal epilepsies: Frontotemporal | 0.6 |
| Other non-lesional focal epilepsies: Occipital | 0.9 |
| Other non-lesional focal epilepsies: Temporal | 30.7 |
| Other non-lesional focal epilepsies: Unspecified | 5.4 |
| Genetic generalised epilepsy: Unspecified | 3.9 |
| Genetic generalised epilepsy: Early onset absence epilepsy | 0.3 |
| Genetic generalised epilepsy: Epilepsy with eyelid myoclonia | 0.3 |
| Genetic generalised epilepsy: Late onset | 4.2 |
| Genetic generalised epilepsy: Childhood absence epilepsy | 2.1 |
| Genetic generalised epilepsy: Childhood absence epilepsy/ Juvenile absence epilepsy overlap | 1.2 |
| Genetic generalised epilepsy: Epilepsy with generalised tonic-clonic seizures alone | 2.7 |
| Genetic generalised epilepsy: Febrile seizures only | 4.8 |
| Genetic generalised epilepsy: Febrile seizures plus/ Genetic epilepsy with febrile seizures plus | 1.8 |
| Genetic generalised epilepsy: Juvenile absence epilepsy | 2.1 |
| Genetic generalised epilepsy: Juvenile myoclonic epilepsy | 6.3 |
| Structural focal epilepsies: Benign tumor | 6.0 |
| Structural focal epilepsies: Malformation: Focal cortical dysplasia | 5.1 |
| Structural focal epilepsies: Malformation: Grey matter heterotopia | 0.6 |
| Structural focal epilepsies: Malformation: Polymicrogyria | 1.2 |
| Structural focal epilepsies: Mixed epilepsy lesions | 0.9 |
| Structural focal epilepsies: Others | 2.1 |
| Structural focal epilepsies: Stroke | 1.8 |
| Structural focal epilepsies: Traumatic brain injury | 2.7 |
| Structural focal epilepsies: Vascular malformation: Cerebral angioma | 4.2 |
| Temporal lobe epilepsy: Periventricular nodular heterotopia | 0.3 |
